# Editorial: Unlocking the potential of health data: interoperability, security, and emerging challenges in AI, LLM, precision medicine, and their impact on healthcare and research

**DOI:** 10.3389/fdgth.2026.1783831

**Published:** 2026-01-30

**Authors:** Eugenia Rinaldi, Rebecca Kush, Stefano Dalmiani

**Affiliations:** 1 Berlin Institute of Health at Charité - Universitätsmedizin Berlin, Berlin, Germany; 2 Catalysis Research, Austin, TX, United States; 3 Fondazione Toscana Gabriele Monasterio per la Ricerca Medica e di Sanita Pubblica, Pisa, Italy

**Keywords:** digital health, health data, interoperability, semantic interoperability, standards

The accelerating digitization of healthcare has positioned health data as a central driver of innovation across clinical care, biomedical research, and public health. The *Frontiers in Digital Medicine* Research Topic “Unlocking the Potential of Health Data: Interoperability, Security, and Emerging Challenges in AI, LLM, Precision Medicine, and Their Impact on Healthcare and Research” brings together nine complementary contributions that collectively explore how health data can be actionable, trustworthy, and equitable knowledge rather than fragmented digital artifacts. Taken together, these manuscripts demonstrate that the promise of data-driven medicine is inseparable from the structural, ethical, and technical conditions under which health data are generated, shared, and analyzed.

A recurring theme across the collection is the foundational role of interoperability. Facile et al. argue that meaningful reuse of health data depends not only on technical connectivity but on semantic interoperability—the shared understanding of data across systems, institutions, and disciplines. They emphasize that without harmonized terminologies, metadata, common data models, and use of robust standards, large-scale health data integration risks producing volume without value. Their work highlights interoperability as a socio-technical challenge that requires planning and coordinated governance, not merely technical standards. This perspective is reinforced by Adams et al., who demonstrate how interoperable data commons infrastructure enables scalable time-series analytics and discovery across clinical trials. Their enhancement of the Gen3 platform illustrates how standardized, interoperable environments are critical for reproducible research and for translating complex datasets into actionable insights for precision medicine. These manuscripts emphasize the value of interoperability not only to providers but to patients.

Interoperability also shapes the quality and sustainability of digital health services. Xia et al. show that in healthcare e-government systems, technical integration directly influences perceived service quality, usability, and trust. Their findings underscore that interoperability has tangible human consequences: when systems fail to communicate effectively, patients and providers experience fragmentation, frustration, and disengagement. This work highlights the importance of aligning backend data integration with front-end user experience to ensure that digital health systems are not only functional but adopted and trusted.

As interoperability expands, concerns about data security and privacy intensify. Georgiou et al. address this challenge by proposing a secure cloud repository architecture for continuous monitoring of patients with mental disorders. Their work demonstrates how privacy-by-design principles can be embedded into systems that collect high-frequency, longitudinal health data, ensuring confidentiality while supporting real-time clinical insight. Rather than framing security as a constraint, they position it as an enabling condition for scalable digital care. Complementing this systems-level approach, Veugen et al. focus on analytical privacy by introducing a secure latent Dirichlet allocation framework. Their study illustrates how advanced machine-learning methods can be adapted to mitigate data leakage risks, reinforcing the idea that security must be integrated into both infrastructure and analytical methodology.

Artificial intelligence emerges across the collection of manuscripts as a powerful but double-edged tool. Van Mierlo et al. explore this tension through their evaluation of an AI-powered data curation and publishing virtual assistant. They show that while AI can substantially improve efficiency and accessibility, its success depends heavily on transparency, explainability, and user trust—particularly when systems are deployed in patient-facing or clinical research contexts. Their findings are especially relevant in light of the growing interest in large language models, which promise to mediate between complex health datasets and human understanding but also raise concerns about data quality, reliability, bias, and accountability particularly when trained on heterogeneous, incomplete, or poorly curated health data.

The importance of context in AI-driven analysis is underscored by Grünewald et al., who demonstrate how secondary use of electronic health record data—such as blood pressure measurements—can be misleading when clinical and situational context is ignored. Their work cautions against the assumption that structured data are inherently self-explanatory and emphasizes the need for contextual metadata to support safe and valid reuse. This insight resonates across the collection, reminding researchers and clinicians alike that health data are produced within specific clinical, social, and temporal environments.

Behavioral and human factors further shape the real-world impact of health data systems. Wang et al. examine determinants of frequent online medical record use and reveal that access alone does not guarantee engagement. Digital literacy, perceived usefulness, and trust significantly influence whether patients actively interact with their health data. Their findings suggest that patient-centered design and education are critical complements to technical interoperability, particularly if digital health tools are to support equitable participation.

At the population level, Anderson et al. demonstrate the power of integrated real-world data through their analysis of COVID-19 patients in critical care settings. By identifying clinically meaningful sub-populations, they show how harmonized datasets can support precision approaches even in acute, resource-constrained environments. Their work exemplifies how data integration, when combined with robust analytics, can translate directly into improved risk stratification and care planning.

Collectively, these nine contributions highlight that precision medicine is not solely an outcome of advanced algorithms or large datasets, but the product of an ecosystem in which interoperable, secure, context-aware, and ethically governed data can be responsibly reused. Several authors caution that without deliberate attention to equity, data-driven approaches risk reinforcing existing disparities. Facile et al. and Van Mierlo et al. both note the potential for bias embedded in AI systems and the value of data standards implemented from the start, while Li et al.'s findings on digital engagement reveal structural inequalities that shape who benefits from digital health innovations.

In synthesis, this Research Topic makes clear that unlocking the potential of health data requires coordinated progress across technical, ethical, and human dimensions. Interoperability must be semantic as well as technical, security must be proactive and embedded, AI must be transparent and accountable, and data reuse must remain grounded in context and equity. The studies collected here move the field beyond abstract enthusiasm toward practical, evidence-based pathways for realizing the promise of digital medicine. As healthcare systems continue to evolve, these insights provide a timely and necessary foundation for building data ecosystems that are not only powerful, but trustworthy and inclusive.

